# Patient perspectives on tablet-based technology to collect risk factor information in primary care

**DOI:** 10.1186/s12875-021-01443-7

**Published:** 2021-05-26

**Authors:** Leanne Kosowan, Alan Katz, Gayle Halas, Alexander Singer

**Affiliations:** 1 Department of Family Medicine, Rady Faculty of Health Sciences, University of Manitoba, Winnipeg, Manitoba Canada; 2 Manitoba Centre for Health Policy and Departments of Community Health Science & Family Medicine, Rady Faculty of Health Sciences, University of Manitoba, 408-727 McDermot Ave., Winnipeg, Manitoba R3E 3P5 Canada

**Keywords:** Risk Factors, Information technology, Primary health care, Primary prevention

## Abstract

**Background:**

Primary care provides an opportunity to introduce prevention strategies and identify risk behaviours. Algorithmic information technology such as the Risk Factor Identification Tool (RFIT) can support primary care counseling. This study explores the integration of the tablet-based RFIT in primary care clinics to support exploration of patient risk factor information.

**Methods:**

Qualitative study to explore patients’ perspectives of RFIT. RFIT was implemented in two primary care clinics in Manitoba, Canada. There were 207 patients who completed RFIT, offered to them by eight family physicians. We conducted one-on-one patient interviews with 86 patients to capture the patient’s perspective. Responses were coded and categorized into five common themes.

**Results:**

RFIT had a completion rate of 86%. Clinic staff reported that very few patients declined the use of RFIT or required assistance to use the tablet. Patients reported that the tablet-based RFIT provided a user-friendly interface that enabled self-reflection while in the waiting room. Patients discussed the impact of RFIT on the patient-provider interaction, utility for the clinician, their concerns and suggested improvements for RFIT. Among the patients who used RFIT 12.1% smoked, 21.2% felt their diet could be improved, 9.3% reported high alcohol consumption, 56.4% reported less than 150 min of PA a week, and 8.2% lived in poverty.

**Conclusion:**

RFIT is a user-friendly tool for the collection of patient risk behaviour information. RFIT is particularly useful for patients lacking continuity in the care they receive. Information technology can promote self-reflection while providing useful information to the primary care clinician. When combined with practical tools and resources RFIT can assist in the reduction of risk behaviours.

**Supplementary Information:**

The online version contains supplementary material available at 10.1186/s12875-021-01443-7.

## Background

There are increasing rates of chronic conditions related to modifiable risks including physical inactivity, smoking, and poor diet [1, 2]. Primary prevention strategies aim to increase physical activity, improve nutrition and decrease substance use to prevent associated morbidity and premature death [1–9]. Individualized primary prevention counseling that considers the social determinants of health is associated with reduced risk behaviour as well as improved mental health and health outcomes [10, 11].

Primary care includes acute care, chronic disease management and services aimed at health maintenance. The Patient Centered Medical Home in the United States and Patient Medical Home in Canada supports team-based primary care [12, 13]. Primary care teams may include physicians, nurses and allied health professionals, who can promote health behaviors and implement primary prevention counseling [10, 12–23]. Primary care counselling can be enhanced by priming the patient to reflect on their risk behaviors prior to their appointment with a clinician [24–26]. Although patient-centered health risk assessments have a positive impact on primary prevention, [24] a recent study found that less than half of patient records had documentation of physical activity in the patient’s chart [27].

Algorithmic information technology to collect, organize and synthesize information about an individual has been employed in patient-directed mobile applications to support health behaviour change [17, 21–24, 28–35]. The Risk Factor Identification Tool (RFIT) is an algorithmic information technology survey designed by our team of researchers and family physicians [22, 23]. RFIT uses previously validated assessment questions and the trans-theoretical model to collect information on a patient’s risk behaviors, and readiness to change target behaviours prior to their annual health review with their physician [8, 32, 36–41]. RFIT when connected to the patient’s Electronic Medical Record (EMR) contributes to a comprehensive record of risk behaviours that can inform personalized approaches to primary prevention. Previously, clinicians and clinic staff found RFIT to be a practical preventative tool in family practice [22, 23]. This study aims to assess the feasibility and integration of the tablet-based RFIT for collection of patient risk factors at primary care clinics, from the patient’s perspective. Analyzing RFIT and interview responses, complemented with the clinic’s experience, this study describes the use of RFIT and resulting primary prevention counselling at family practice clinics.

## Methods

### The RFIT

Using a response-based algorithm, the tablet-based RFIT captures patient risk behaviours. Questions incorporated motivational interviewing and health coaching modalities [22]. RFIT collects basic demographic data and asks patients about their physical activity, diet, smoking, alcohol use, self-perceived health and self-reported low-income (i.e. do you find yourself running out of money for food or shelter; do you have trouble paying for medication) (Additional file 1: Appendix A) [37–42]. Readiness to change a risk behaviour is assessed using the trans-theoretical model [32, 36, 41]. Using the Ocean App developed by CognisantMD RFIT responses are integrated into the EMR (Additional file 1: Appendix B) [43].

### Recruitment

In Canada necessary health care is provided in hospital and community settings through single-payer, publicly funded insurance. Primary care is usually the first contact with the health system. Eight family physicians from two primary care clinics in Manitoba, provided informed consent prior to offering RFIT to their patients attending an annual health review. A health review is an appointment focused on health maintenance and presents an opportunity for primary prevention counselling. Patients were offered the opportunity to use RFIT by the clinic reception staff. Prior to answering the RFIT questions, the tablet provided patients with information on RFIT and this study. Patients provided informed consent to participate on the tablet. Patients could also choose to complete RFIT but not participate in this study. After completing RFIT the patient’s responses were automatically transferred into an encounter note in the EMR, providing initial screening information for the physician to use and integrate into clinical care (Additional file 1: Appendix B). Following completion of RFIT the tablet asked patients who provided informed consent if they could be contacted for an interview about their experience using RFIT. Patients who provided their name and phone number were contacted for an interview. Informed consent was obtained orally for the interview as approved by the Health Research Ethics Board.

### Data collection

Patient RFIT responses were extracted from the Ocean online platform upon study completion. This included patient demographics, risk behaviours, readiness to change, and self-perceived health and social circumstances. Patients also participated in one-on-one telephone interview which assessed patient perceptions of the tablet, RFIT, and primary prevention discussions (Additional file 1: Appendix C).

### Analysis

Quantitative data included user logs, audit reports, patient RFIT responses, and patient interviews (multiple-choice and short-answer). Descriptive statistics including frequency, mean, standard deviation (SD) and range characterized the patients and their risk behaviours. Chi-square analyses assessed change in the number of risk behaviour discussions (e.g. physical activity, diet, smoking, alcohol, poverty) following the use of RFIT. Statistical analysis was generated using SAS® software, Version 9.4 of the SAS System for [Windows × 64] Copyright © [2002–2012] SAS Institute Inc. SAS and all other SAS Institute Inc. product or service names are registered trademarks or trademarks of SAS Institute Inc., Cary, NC, USA.

Three open-ended questions were asked during the patient interviews: (1) How useful do you feel the tablet RFIT survey was for you?; (2) Do you feel it made a difference to the discussion you had with your primary care provider?; (3) Do you have any additional comments about the tablet or the RFIT survey? Responses to these questions were transcribed. Transcripts were coded and codes were categorized into common themes. Two team members reviewed the codes and quotes within each theme. Five themes emerged from the open-ended interview questions: (1) well-designed, helpful tool, (2) impact on patient-provider interaction, (3) self-reflection, (4) useful to clinicians, (5) concerns and suggested improvements.

## Results

Of the 207 patients who started RFIT, 179 (86%) completed RFIT. On average patients took 11 min to complete RFIT. Approximately 3–4% of patients declined RFIT due to concerns regarding privacy, technology, appointment delays and language barriers.

Eighty-six patients (48.0%) who completed RFIT were interviewed. Patients interviewed were similar to patients who had completed RFIT. However, patients interviewed were older on average than patients who completed the tool (57.5 vs. 51.4 p = 0.001). They were also less likely to be employed (59.2% vs. 50.6% p = 0.04) (Table 1). Interviewed patients frequently reported that they were retired (n = 31/39.2%). Among the patients interviewed 95.0% reported that this was their regular healthcare provider and 80.0% had seen this clinician for more than 5 years.

**Table 1:** Patient responses to the RFIT on the tablet

Variable	RFIT Tablet-Survey *N* = 207	Patient Interview *n* = 86	*p*-value
**Patient Characteristics**			
Rural Clinic	56.0% (116/207)	62.8% (54/86)	0.099
Mean age in years (SD)	51.4 (15.2)	57.5 (15.0)	**0.001**
Female Patient	77.5% (145/187)	79.3% (65/82)	0.617
Married or common-law	-	76.3% (61/80)	-
Post-secondary education	-	64.6% (51/79)	-
Mean BMI (SD)	27.6 (7.99)	27.2 (7.05)	0.73
**Health Status**			
Excellent, very good or good Self-Perceived health	87.9% (160/182)	84.2% (64/76)	0.1
Self-perceived health unchanged from last year	70.6% (127/182)	68.1% (49/72)	0.68
Average number of visits per year (SD)	-	3.32 (3.66)	-
Health condition of interest^1^	-	61.3% (49/80)	-
**Risk Factors**			
Self-reported smoker	12.1% (22/182)	11.3% (9/80)	0.76
Self-reported healthy diet	78.8% (145/184)	75.3% (61/81)	0.3
CAGE Flag for alcohol consumption	9.3% (17/182)	10% (8/80)	0.79
Less than 150 min of PA	56.4% (102/181)	54.5% (42/77)	0.67
Employed	59.2% (106/179)	50.6% (40/79)	**0.04**
Poverty	8.2% (6/73)	12.8% (5/39)	0.13

Questions on RFIT and in the patient interviwe were not mandatory; patients could choose to answer or not answer a question

^1^Health conditions of interest include: pre-diabetes, diabetes, high blood pressure, heart disease, liver disease, arthritis, kidney disease, cancer, asthma, lung disease, osteoporosis, thyroid disorders

### Well-designed, helpful tool

Most patients provided positive feedback regarding their experience with RFIT. One patient explained, *“[RFIT was] Good. It did not take long to complete. Asked questions so the doctor did not have to*”. Another patient stated, *“It was great. It was very convenient and a pleasant surprise. Simple and easy to use, easy to read…”.*

In addition, 98% of interviewees reported that the tablet-based RFIT was well-designed, with only 8.8% requiring assistance from reception staff. For example, one patient required assistance to select a response using a drop-down menu. The majority of participants (96.3%) had some experience with computers. However one patient shared, *“It was easy to use. The questions were good. I use a computer very little but was able to use this tablet for this survey*.” Similarly, clinic staff found that most patients did not share any concerns about RFIT. Patients described RFIT as an efficient method for risk behaviour assessment. “*I thought it was a good idea as soon as I saw it. I am waiting anyways. And it saves time for the doctor because she does not have to ask as many questions…*”.

### Impact on patient-provider interactions

There was mixed feedback on the influence of RFIT on the patient-provider interaction. Twenty-four patients felt that their annual health review was similar the previous year. *“[My doctor] normally goes through the same list [of risk behaviours]. So these topics were not out of ordinary. But we did not need to discuss them. We might have discussed them if my answers were different*.”

Only 15% of patients reported that completing the RFIT made a difference to the discussion they had with their family physician. “*[My doctor] looked at my answers and made comments. It was a quick way for her to review these areas instead of asking me all the questions.”*

**Fig. 1:**
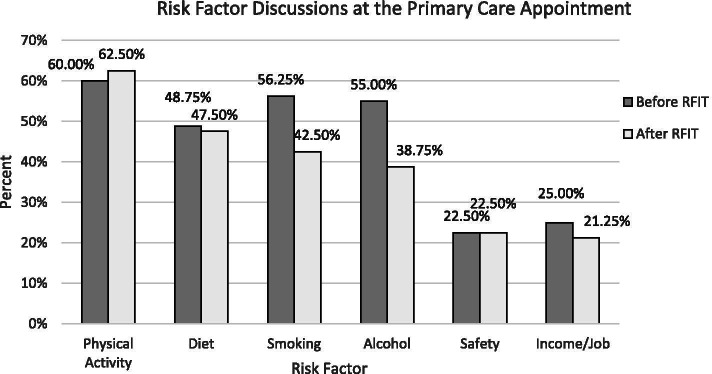
Risk factor discussions at the primary care appointment

Risk behaviour discussions largely focused on physical activity and infrequently addressed safety (i.e. sunscreen, hats, helmets) or income (Fig. 1)., Patients reported few discussions about alcohol use and smoking (Fig. 1). There were patients who did not discuss any risk behaviours at an appointment prior to, or after, the completion of RFIT. A small group of patients wanted to discuss one or more of the risk factors that was not discussed during their appointment.

Almost half on the respondents felt that RFIT did not influence their appointment. Patients explained that they were unsure if the RFIT answers were reviewed by their physician. One patient stated, *“[RFIT] could have [made a difference]. I think they (the doctor) might have asked more questions if I did not respond the way I did.”* Some patients had an acute care concern that they wanted to discuss during their appointment. *“I don’t know if it (RFIT) added to the discussion… I went in focused on one thing and it turned into a larger health review… and included discussions on physical activity and diet. So maybe it did…”* And other patients felt that RFIT did not make a difference to their patient-provider interaction. *“My health care provider knows me and my family very well. She regularly asks me about physical activity and nutrition, and knowns that there are no concerns with smoking, alcohol or income”.*

Patients thought that the RFIT would be helpful for new patients or patients that infrequently visited their clinician. One patient explained, *“If I was new it would be a great starting point to have this discussion to introduce yourself and your situation to your provider.”*

### Self-reflection

A quarter of the patients thought RFIT was a great tool for self-reflection. One patient explained, “*I liked the survey a lot. It made me think of questions and areas that I do not usually think about*.”

Patients indicated increased awareness of these behaviours and self-directed changes they could make. *“The survey reminds you. It gets you thinking about these things. I did not make any drastic changes since completing the survey, but it did make me more aware.”*

### Useful to clinician

Patients thought RFIT was a useful tool for the physician. For example one patient stated, “[*RFIT is] important. It can save the doctor time.*” While another explained, *“This is very beneficial. Time management is very important in health. This provides cues for the patient and provider… When and where to focus…”.*

### Concerns and suggested improvements

Patients suggested a variety of ways that RFIT could be improved including formatting, stylistic changes, and follow-up suggestions. For example patients suggested, inclusion of optional open-ended questions, additional tablet explanation from the clinic, and assistance in calculating the minutes of physical activity.

*“I felt limited in my ability to answer some of the questions. Some questions were yes or no. I could not explain… I could not say I always eat whole grain, but I usually do.”*

Some patients hoped that in the future there would be additional follow-up and support for implementing and maintaining healthy behaviours.

*“I would like more follow-up. Follow-up would have been nice. Something other than telling me to eat healthy and go to the gym. It is not working for me. I have social issues and have trouble getting myself to go to the gym… [I] have been battling weight issues all my life. The gym does not help… I think my doctor thinks I am just getting old. Or I just need to be more active. But it is more than that...”*

Very few participants were concerned about privacy. Most patients agreed with the sentiment that *“[they felt] comfortable because they trusted their physician to protect their information.”*

## Discussion

This study implemented an algorithmic tablet-based tool in primary care that enhanced the collection of risk factor information. Implementing RFIT within primary care clinics provided information to the physician and facilitated immediate counseling following survey completion. Overall, the use of the tablet-based RFIT was well-received by patients of varying ages and socio-demographic backgrounds. The majority of patients had previous experience with a computer or tablet, however even patients without this experience were able to complete RFIT. RFIT presented an opportunity for patients to reflect on their risk behaviours, while providing information to the physician. However, there were some suggestions for improving RFIT and its implementation.

In previous evaluations RFIT’s use of information technology for the collection of risk factor information was favorably reviewed by clinicians and clinic staff [22, 23]. In this study, patients felt that the tablet-based survey was a well-designed tool for the collection of risk behaviour information. Patients were happy to occupy their time in the waiting room and very few patients required assistance with RFIT. Similar assessment tools, such as Case Finding Help Assessment Tool (CHAT) implemented in New Zealand, have been reviewed favorably by both clinicians and patients [21, 34, 35, 44–48]. Although previous studies have suggested assessment tools should focus on psychosocial factors, this study found that tailoring the tool to the appointment type as well as population are important [34, 35]. Revisions of CHAT to support the collection of lifestyle and mental health factors from sub-populations have been favorably evaluated [46–48]. Adjusting the RFIT questions and EMR integrated responses to the appointment type and patient characteristics can ensure specific details required to support behaviour change are available to the physician. CHAT identified that 35% of responders required assistance with a topic identified on the assessment tool [44]. We found that only 15% of patients reported that RFIT led to the discussion of a risk behaviour. Interestingly, while 9% of patients were flagged for alcohol use concerns, slightly more than half of these patients did not want to discuss their alcohol consumption with their physician.

Seventy-eight percent of patients we interviewed had been asked about one or more risk behaviours during the patient’s previous health review. Smoking is often the most common risk behaviour discussed [16]. However, counseling on physical activity (60.0%) was more common in this study. Lindeman et al. found that when physician encounters were reviewed the amount of physical activity documented ranged from 0.4 to 87.8% depending on the physician, and that the majority of patients with documentation of physical activity were patients with a chronic disease [27]. In this study 61.3% of patients had a chronic disease which may have contributed to the majority of patients having discussed risk behaviours in the previous year.

Patients in this study perceived RFIT as a beneficial, user-friendly tool. Patient suggestions about the tool sometimes contradicted design elements intended to make RFIT more user friendly for example avoiding open-ended questions. Patient’s positive assessment of the RFIT and resulting self-reflection supports the use of RFIT in primary care practices. Patient’s input in primary prevention counselling promotes a therapeutic alliance and is important in information exchange [49]. However, the use of RFIT responses by clinicians was variable. Patients reported not receiving counselling for a risk factor that they felt should have been discussed. Offering RFIT to new or infrequent patients may encourage additional review and better inform patient-clinician discussions. Additionally, although this study provided a list of resources to support behaviour change, clinicians and patients reported that they did not feel supported to make changes. Brief discussions and counselling in primary care can positively influence patients’ health habits including prevention activities (e.g. increasing physical activity) and health-promotion behaviours [14, 50]. Additional supports such as prescribing healthy behaviours and referral for more extensive interventions can provide comparative benefits for risk behaviours through connections with allied health professionals [50–53]. Interventions must be supported by professional and personal relationships, attend to a patient’s health and social needs, and be designed based on individual patients interests to create new habits for health behaviours [6, 50, 54].

### Limitations

Our findings are not based on a representative sample of patients in Manitoba. Patients who regularly attend a health review appointment are more likely to already receive primary prevention counselling. Patients who regularly visit their primary care provider may be different from patients who do not regularly have an annual health review. Patients who did not consent to participate in this study may be different from those who consented. Our findings are based on implementation in family physician offices in a publicly funded health care system and may not be generalizable to other clinical environments.

## Conclusions

RFIT assisted in the collection of risk factor information from patients of varying ages and demographics. However, RFIT may be particularly useful for new patients or patients lacking continuity in their care. This study demonstrated that RFIT promotes patient self-reflection however, practical tools and resources are needed by providers and patients so interventions can be tailored to support reduction in risk factors. Future implementation of RFIT should be complemented with a team of allied health professionals prepared to support referrals from primary care.

SAS and all other SAS Institute Inc. product or service names are registered trademarks or trademarks of SAS Institute Inc. in the USA and other countries. ® indicates USA registration.

## Supplementary Information

**Additional file 1:** **Appendix A.** RFIT Computerized Risk Behaviour Assessment. **Appendix B.** RFIT EMR Encounter Not Example. **Appendix C.** Risk Factor Identification Tool (RFIT) Patient Telephone Interview Guide

## Data Availability

The datasets generated and/or analyzed during the current study are not publicly available due to the confidential nature of the data governed by PHIA legislation, but are available from the corresponding author on reasonable request.

## Abbreviations

RFIT: Risk Factor Identification Tool
EMR: Electronic Medical Record
SD: Standard Deviation
CHAT: Case Finding Help Assessment Tool
